# The spike-specific TCRβ repertoire shows distinct features in unvaccinated or vaccinated patients with SARS-CoV-2 infection

**DOI:** 10.1186/s12967-024-04852-1

**Published:** 2024-01-07

**Authors:** Eleonora Vecchio, Salvatore Rotundo, Claudia Veneziano, Antonio Abatino, Ilenia Aversa, Raffaella Gallo, Caterina Giordano, Francesca Serapide, Paolo Fusco, Giuseppe Viglietto, Giovanni Cuda, Francesco Costanzo, Alessandro Russo, Enrico Maria Trecarichi, Carlo Torti, Camillo Palmieri

**Affiliations:** 1 Department of Experimental and Clinical Medicine, University “Magna Graecia”, Viale Europa, 88100 Catanzaro, Italy; 2 Department of Medical and Surgical Sciences, Chair of Infectious and Tropical Diseases, University “Magna Graecia”, 88100 Catanzaro, Italy; 3 Interdepartmental Centre of Services, University “Magna Graecia”, 88100, Catanzaro, Italy

**Keywords:** SARS‐CoV‐2, COVID‐19, TCRβ repertoire, Breakthrough infection

## Abstract

**Background:**

The evolving variants of SARS-CoV-2 may escape immunity from prior infections or vaccinations. It’s vital to understand how immunity adapts to these changes. Both infection and mRNA vaccination induce T cells that target the Spike protein. These T cells can recognize multiple variants, such as Delta and Omicron, even if neutralizing antibodies are weakened. However, the degree of recognition can vary among people, affecting vaccine efficacy. Previous studies demonstrated the capability of T-cell receptor (TCR) repertoire analysis to identify conserved and immunodominant peptides with cross-reactive potential among variant of concerns. However, there is a need to extend the analysis of the TCR repertoire to different clinical scenarios. The aim of this study was to examine the Spike-specific TCR repertoire profiles in natural infections and those with combined natural and vaccine immunity.

**Methods:**

A T-cell enrichment approach and bioinformatic tools were used to investigate the Spike-specific TCRβ repertoire in peripheral blood mononuclear cells of previously vaccinated (n = 8) or unvaccinated (n = 6) COVID-19 patients.

**Results:**

Diversity and clonality of the TCRβ repertoire showed no significant differences between vaccinated and unvaccinated groups. When comparing the TCRβ data to public databases, 692 unique TCRβ sequences linked to S epitopes were found in the vaccinated group and 670 in the unvaccinated group. TCRβ clonotypes related to spike regions S135-177, S264-276, S319-350, and S448-472 appear notably more prevalent in the vaccinated group. In contrast, the S673-699 epitope, believed to have super antigenic properties, is observed more frequently in the unvaccinated group. In-silico analyses suggest that mutations in epitopes, relative to the main SARS-CoV-2 variants of concern, don’t hinder their cross-reactive recognition by associated TCRβ clonotypes.

**Conclusions:**

Our findings reveal distinct TCRβ signatures in vaccinated and unvaccinated individuals with COVID-19. These differences might be associated with disease severity and could influence clinical outcomes.

*Trial registration*: FESR/FSE 2014–2020 DDRC n. 585, Action 10.5.12, noCOVID19@UMG.

**Supplementary Information:**

The online version contains supplementary material available at 10.1186/s12967-024-04852-1.

## Background

The severe acute respiratory syndrome coronavirus 2 (SARS-CoV-2) pandemic requires continuous attention focused on the epidemiological, virological, and immunological characteristics of the evolving variants of concern (VoCs). Distinct antigenic characteristics of VoCs could allow the virus to escape from immunity generated through previous infection and/or vaccination, eluding the protective immunity against re-infections and severe disease courses [1]. For this reason, it is important to evaluate as many variables as possible that can influence the adaptive immunity against the infection, as well it is relevant to understand how these variables change in relation to the evolution of the epidemiological, virological, and immunological landscape.

SARS-CoV-2 infection and mRNA vaccination were shown to induce spike (S) specific T cells that can recognize and eliminate infected cells [2–5]. These S specific T cells can largely, albeit not completely, tolerate the amino acid mutations that characterize the different VoCs, including Delta and Omicron [6, 7], and respond to the viral variants. Such T cell cross-reactivity against VoCs mutant peptides contributes to protection against severe disease, hospitalization, and death, even if the neutralizing antibody response is partially compromised [8–10]. However, it is important to note that the degree of T cell cross-reactivity may vary among individuals, and the overall effectiveness of current vaccines against new emerging VoCs may be reduced.

The specificity of T cell response is determined by the T cell receptor (TCR), which is produced through a stochastic process of somatic recombination that combines the unique V, D and J gene segments of the TCRα and TCRβ genes (D segments only for TCRβ), resulting in huge range of TCRs with an incredibly diverse antigenic specificity. The third complementarity‐determining regions (CDR3s) localized within the TCRα and TCRβ chains are the most hypervariable regions and takes part to direct peptide recognition. Recent advances in TCR sequencing technologies and bioinformatic analysis allow the characterization of the TCR repertoire, that is the collection of diverse and unique TCRs within an individual's immune system, with great throughput and efficiency [11]. Analysis of the TCR repertoire provides summary indices of the diversity and clonality of T-cell responses that may be associated with the clinical evolution of a disease, as well as it allows extensive profiling of T cell specificities, despite the complexity of these responses across individuals and groups [11–13].

Recent studies found that the TCR repertoire of SARS-CoV-2 specific T cells is highly diverse, which is important for recognizing and clearing the virus [2, 14]. Moreover, it was observed that COVID-19 patients affected by severe disease have a restricted TCR repertoire and an increased frequency of public TCRs, suggesting that TCR diversity may play a role in determining disease outcomes. The mRNA vaccines elicited a diverse TCR repertoire, indicating a robust T cell response and supporting the efficacy of SARS-CoV-2 vaccines in generating an adaptive immune response [10]. These analyses also identified immunodominant TCRs associated with S-specific CD8 + T cell responses [10, 15]. While these studies demonstrated the utility of T-cell repertoire analysis in identifying conserved and immunodominant peptides with cross-reactive potential among VoCs, predicting disease severity, and informing treatment strategies, they also emphasized the need to extend TCR repertoire analysis to different clinical scenarios.

In this study, we analyzed the TCR repertoires generated during natural SARS-CoV-2 infection in unvaccinated patients or hybrid immunity (infection-induced and vaccine-induced immunity), highlighting distinctive S-specific TCR profiles between the groups.

## Methods

### Study design

An observational longitudinal study was performed on 14 consecutive patients tested positive for SARS-CoV-2 with mild to severe COVID-1912 from January 24th to July 7th, 2022. The characteristics of the participants are summarized in Table 1. The study was conducted according to the standards of the Declaration of Helsinki revised in 2008 (World Medical, 2013), and was approved by the ethical committee of the Calabria Region (Protocol Reference: FESR/FSE 2014–2020 DDRC n. 585, Action 10.5.12, noCOVID19@UMG). Written informed consent was obtained from all the participants before moAbs administration and blood samples collection for the purpose of this study.

**Table 1:** Patient characteristics

Patient ID	Gender	Age	Diagnosis	Vacc n. doses	Severity^a^	Respiratory status	Relevant risk factors^b^	HLA^c^
NV1	F	28	PCR	0	Mild	Spont.breath	i.d	A*01:01/02:01 B*35/39 DRB1*08/11 DQB1*03/04
NV2	F	83	PCR	0	Moderate	Sup_O_2_	DM	n/a
NV3	M	80	PCR	0	Severe	Sup_O_2_	DM	A*02:01 DRB1*04 DQB1*03
NV4	M	75	PCR	0	Mild	Spont.breath	DM	A*02:01 B*35/44 DRB1*11/04 DQB1*03/06
NV5	M	55	PCR	0	Severe	Sup_O_2_	i.d	A*02:01 DRB1*04 DQB1*03
NV6	M	49	PCR	0	Mild	Spont.breath	i.d	A*02:01/24:02
V1	M	70	PCR	3	Mild	Spont.breath	DM, obesity	A*02:01/11:01 DRB1*04 DQB1*03/06
V2	M	73	PCR	3	Mild	Spont.breath	diabetes	n/a
V3	M	58	PCR	3	Mild	Spont.breath	i.d	A*02:01 B*35 DRB1*04 DQB1*03
V4	M	64	PCR	3	Mild	Spont.breath	i.d	n/a
V5	M	56	PCR	3	Mild	Spont.breath	i.d., obesity	A*02:01 B*35/44 DRB1*04
V6	M	51	PCR	3	Mild	Spont.breath	none	n/a
V7	F	69	PCR	3	Mild	Spont.breath	none	A*01:01/02:01 DRB1*08/11 DQB1*03/04
V8	M	37	PCR	3	Mild	Spont.breath	i.d	A*02:01/03:01 B*27/40

^**a**^Severity according to COVID-19 Treatment Guidelines Panel. Coronavirus Disease 2019 (COVID-19) Treatment Guidelines [23]

^b^i.d.—primary/acquired immunodeficiency; DM diabetes

^c^n/a—not available

### PBMCs purification and *in-vitro* T-cell expansion

Peripheral venous blood was collected in EDTA vacutainer tubes, and peripheral blood mononuclear cells (PBMCs) were isolated by density gradient isolation using Ficoll-Paque (Merck, KGaA, Darmstadt, Germany), according to the manufacturer’s instructions. The isolated PBMCs were immediately divided into two aliquots, one subjected to RNA extraction for subsequent TCR sequencing analysis, the other used for the T cell expansion procedure. For *in-vitro* T-cell expansion, 10^6^ PBMCs were seeded cultured with Advanced RPMI Medium 1640 supplemented with 2% human serum, 2 mM L-Glutamine, and 100 U/penicillin/streptomycin in 24-well. Then cells were stimulated for 12 days with 2 μg/mL of SARS-CoV-2 (S-pool) and 50 U/mL rIL-2 and incubated in a humidified CO_2_ incubator at 37 ℃ changing medium with fresh S-pool and rIL-2 every 2 days [16, 17]. The S-pool consisted in 15-mer peptides that overlapped by 10 amino acids and spanned the entire protein sequence of the S protein of SARS-CoV-2 (Uniprot_ID = P0DTC2) [17].

### Enzyme-linked immunoSpot assay

Enzyme-linked immunoSpot (ELISPOT) path kit (cod.3420-4AST-P1-1, Mabtech, Sweden) was used for the enumeration of PBMCs secreting interferon gamma (IFNγ) in response to S-pool, according to manufacturer's instructions. Spots corresponding to stimulated cells secreting IFNγ) were counted by an immunoSpot plate analyzer (BIOREADER3000; Bio-Sys, Germany). The IFNγ-ELISPOT data were reported as stimulating forming units × 10^6^ PBMCs (SFU/10^6^), which was calculated for each PBMC sample by subtracting spots of the unstimulated wells from the spots of the peptide-stimulated wells and normalizing to 10^6^ PBMCs [17].

### TCR sequencing

TCRβ libraries for NGS sequencing were prepared using the Oncomine™ TCR Beta‑LR Assay (ThermoFisher), according to the manufacturer’s protocol. In detail, RNA from PBMCs samples was isolated using the Purelink RNA Mini kit (Thermo Fisher Scientific, Milan, Italy), reverse transcripted through the InvitrogenTM SuperScript IV VILO Master Mix (ThermoFisher Scientific). Libraries were prepared using the TCR beta-LR Assay Kit (ThermoFisher Scientific), which consists of Multiplex AmpliSeq primers target the framework region 1 (FR1) and costant (C) regions of the TCRβ producing a 330 bp amplicon which covers the entire variable gene and the CDR3 region. Libraries were produced for n = 14 individuals for a total of n = 28 samples, i.e. 2 samples points for each individual (pre and post S-specific TCR expansion). Libraries preparation was performed manually according to the Ion AmpliSeq Kit for Chef DL8 (ThermoFisher Scientific). The final concentration of manually prepared cDNA libraries was determined on the Agilent 2200 System by the Agilent High Sensitivity DNA Assay (Agilent Technologies, Santa Clara, CA, USA), following manufacturer’s recommendations. Barcoded libraries were diluted to 25 pM and then loaded onto the Ion ChefTM Instrument (ThermoFisher Scientific) for emulsion PCR, enrichment, and loading onto the Ion S5 530 Chip. Post-sequencing run analysis was performed by the Ion Torrent Suite Software. V, D and J-segment alignment, CDR3 identification and assembly of reads into clonotypes were performed with MiXCR (v.4.1.2) with the built-in preset pipeline “Oncomine™ TCR Beta‑LR Assay” [18].

### TCR repertoire analysis

TCR repertoire analysis was mostly performed using the Immunarch R package [19]. The diversity of TCR repertoires was evaluated by the Gini, Gini-Simpson and d50 diversity coefficients. The Gini coefficient measures the inequality in the frequency distribution of clonotypes, with values close to zero expressing full equality of clonotype frequencies, while a Gini coefficient of 1 reflects maximum inequality between clonotype frequencies, such as the co-presence of hyper-expanded clonotypes and rare clonotypes. The d50 coefficient calculates the minimum number of distinct clonotypes amounting to greater than or equal to 50 percent of a total of sequencing reads obtained following amplification and sequencing. The Gini-Simpson index is the probability of interspecific encounter, i.e., probability that two entities represent different types. Top10, rare and hyper-expanded clonotypes abundance were calculated through the repClonality function of Immunarch. The distribution of Vβ gene segments between COVID-19 groups was performed using the Gene Usage Analysis tool of Immunarch.

### In-silico analysis

The GLIPH2 algorithm of turboGLIPH R package was used for clustering of TCRβ sequences [12]. The minimum cluster size parameter was set to 8. The mapping of S epitopes associated with clonotypes was carried out by recognizing in the repertoires the TCRβ experimentally associated with S epitopes (public databases MIRA [20] and VDJdb [21]), as well as inferring the S-specificity of clonotypes using the GLIPH algorithm.

The pEptide tcR matchinG predictiOn (ERGO) tool [22] was used to classify TCR-peptide binding affinity toward S peptides. The CDR3 of TCR is the major determinant of T cell specificity. The complete list of viral peptides and CDR3 sequences used as input on ERGO is reported in Table 3, respectively. As output, ERGO produces a binding probability score (maximum value 1 if the TCR and the peptide bind and 0 otherwise).

### Statistical analysis

Statistical tests were selected based on appropriate assumptions with respect to data distribution and variance characteristics;* p* values < 0.05 were considered statistically significant. Statistical significances are reported in the figure and/or the figure legend. Statistical tests were performed with GraphPad PRISM software 9.3 (GraphPad Software, La Jolla, CA, USA).

## Results

### Characteristics of the enrolled patients and donors

We enrolled 14 consecutive patients tested positive for SARS-CoV-2 with mild to severe COVID-19 [23] from January 24th to July 7th, 2022. At that time the Omicron variants were predominant in our setting in Calabria region, Southern Italy [24]. Patients with mild COVID-19 symptoms for a maximum of 7 days who did not require oxygen support presented to the center dedicated to early therapies for COVID-19 [25] to receive neutralizing monoclonal antibodies (moAbs) or antivirals (i.e., remdesivir, nirmatrelvir/ritonavir or molnupiravir). Patients with moderate or severe COVID-19 were admitted to hospital. Blood samples for the purpose of this study were collected at least 24 h before starting treatment with moAbs or antivirals. Among these patients, those who did not receive any doses of the approved vaccines were categorized as “not vaccinated” (NV), while those who received at least two doses of BNT162b2 mRNA COVID-19 vaccine were included in the “vaccinated” (V) group [26]. Immunocompromised patients were defined as those affected by onco-hematological diseases, primary/acquired immunodeficiency, systemic inflammatory diseases, or those who received rituximab, methotrexate and/or other immunosuppressive drugs. The characteristics of the participants are summarized in Table 1.

### Diversity and clonality of COVID-19 TCRβ repertoires

**Fig. 1:**
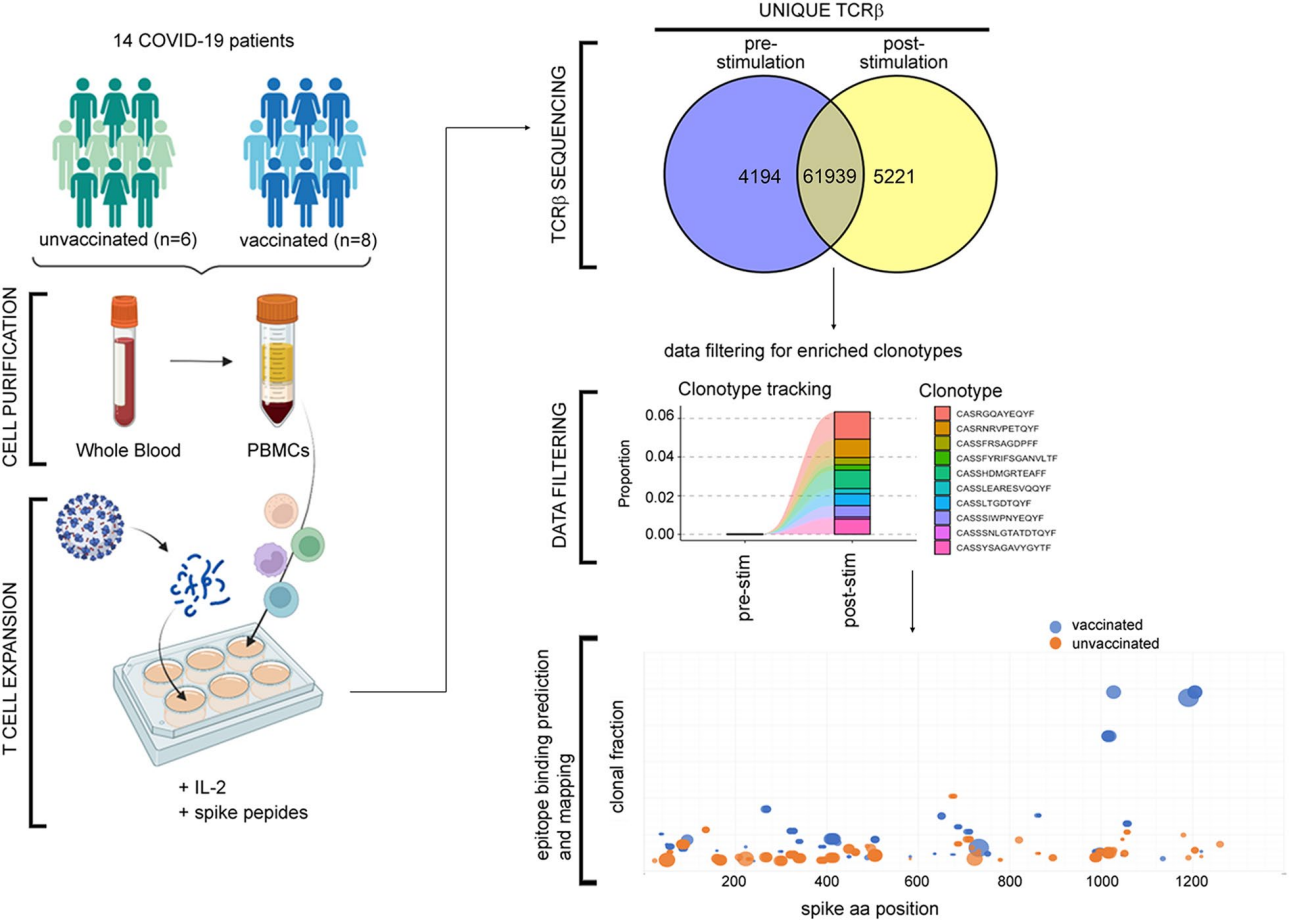
Study workflow. The PBMCs from a whole blood venous sample of COVID-19 patients were divided into two aliquots. One aliquot was directly subjected to TCRβ sequencing (pre-stimulation repertoires). The other aliquot was first stimulated with S-peptides pool, in the presence of IL-2 for 12 days, to promote the expansion of S-specific cells, and then was also subjected to TCRβ sequencing (post-stimulation repertoires). TCRβ repertoire analysis involved a filtering step to exclude confounding sequences not shared between pairs of pre- and post-repertoires. The mapping of S epitopes associated with clonotypes was carried out by recognizing in the repertoires the TCRβ experimentally associated with S epitopes (public databases MIRA [20] and VDJdb [21]), as well as inferring the S-specificity of clonotypes using the GLIPH algorithm [12]

We performed TCRβ sequencing of PBMCs samples both before (pre-stimulation TCRβ repertoires) and after (post-stimulation TCRβ repertoires) T cell enrichment following in vitro 12-day stimulation with a pool of S peptides (Fig. 1). Collectively, we obtained 66133 and 67160 distinct clonotypes (TCRβ with unique CDR3 amino acid sequence) from the pre- and post-stimulation TCRβ repertoires (Fig. 1). Information on TCRβ repertoires characteristics is reported in Table 2 and Supplemental data.

**Table 2:** Information on immune repertoire sequencing and analysis

Patient ID	n_sequence pre-stimul	n_cln^a^ pre-stimul	n_sequence post-stimul	n_cln post-stimul	cln in MIRA^b^	Spike_seq% (pre)^c^	Spike_seq% (post) ^c^
NV1	247282	6409	383272	7742	354	0.0569	0.3942
NV2	273882	5729	323193	5818	151	0.0157	0.1695
NV3	261658	819	265033	782	18	0.0021	0.0141
NV4	257575	13102	301684	14964	366	0.0167	0.1625
NV5	219148	1043	220453	971	16	0.0002	0.0066
NV6	251356	3034	257134	2954	100	0.0027	0.0250
V1	224835	9705	262842	9398	234	0.0190	0.1607
V2	254176	15466	278062	17213	262	0.0097	0.0954
V3	308290	1327	417838	1302	49	0.0258	0.2913
V4	294426	5136	349308	5136	238	0.0105	0.1746
V5	259694	7373	274769	8288	176	0.0072	0.0610
V6	257651	1197	270071	1196	34	0.0070	0.0511
V7	259800	938	262788	900	32	0.0012	0.0126
V8	229324	1054	233338	1048	17	0.0025	0.0191

^a^ n_cln—number of unique CDR3β amino acid sequences

^b^ Number of clonotypes shared with the MIRA/Adaptive dataset

^c^ Fraction of repertoire’ sequences relative to S-specific clonotypes

**Fig. 2:**
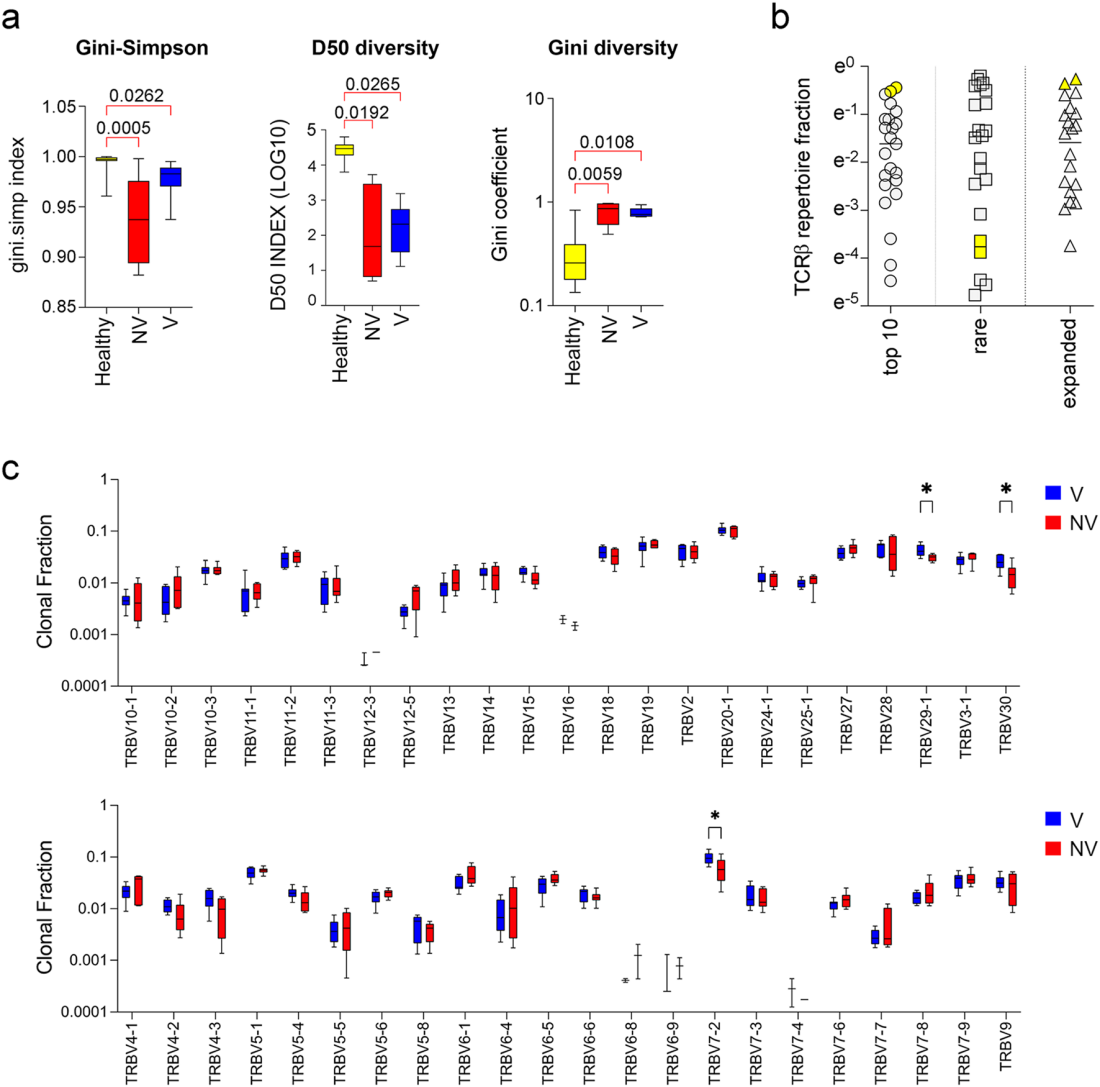
TCRβ repertoires diversity and clonality of COVID-19 and healthy groups. **a** For Gini-Simpson, D50 and Gini diversity significance see the Methods section. Statistical comparison was performed by Kruskal–Wallis test. **b** TCR repertoire fraction of the top 10, rare, and hyper-expanded clonotypes for all repertoires. Top 10 clonotype refers to the 10 most abundant clonotypes from each repertoire; rare and hyper-expanded refer to clonotype with a frequency less than 10^–5^, or greater than 10^–2^, respectively. The position of the value observed for the patient who experienced severe COVID-19 is highlighted in yellow. **c** Comparison of Vβ usage between V and NV COVID-19 groups. Statistical comparison was performed by Mann Whitney test

We first analyzed the diversity and clonality of pre-stimulation COVID-19 TCRβ repertoires, as compared to a control TCRβ dataset obtained from PBMCs sample of healthy individuals (n = 14) from the TCRB-V4b Control Database [27], that matched for age, gender, and ethnicity with our COVID-19 cohort. To rigorously evaluate the diversity of repertoires, we utilized TCRβ repertoire diversity and clonality estimators. Both the Gini-Simpson and D50 diversity indices were significantly lower in the COVID-19 groups compared to the control group, indicating less diverse COVID-19 repertoires (Fig. 2a). Conversely, the Gini coefficient was significantly higher in the COVID-19 groups than in the control group, indicating greater inequality of clonotype frequency (Fig. 2a). Overall, these TCRβ repertoire estimators demonstrated that COVID-19 TCRβ repertoires had higher clonality and lower diversity than the repertoires of healthy subjects, consistent with the expected clonal expansion typical of a cellular response to viral antigens. Notably, the two patients who developed severe COVID-19 exhibited very narrow true diversity and high clonality (Fig. 2b), consistent with previous studies [15]. We also compared the distribution of Vβ gene segments between COVID-19 groups. In the V group, the TRBV7-2, TRB29-1, and TRBV30 Vβ gene segments showed a significantly higher clonal fraction than those observed in the NV (Fig. 2c).

### In vitro expansion of S-specific T cells was equally efficient in PBMCs from vaccinated patients

**Fig. 3:**
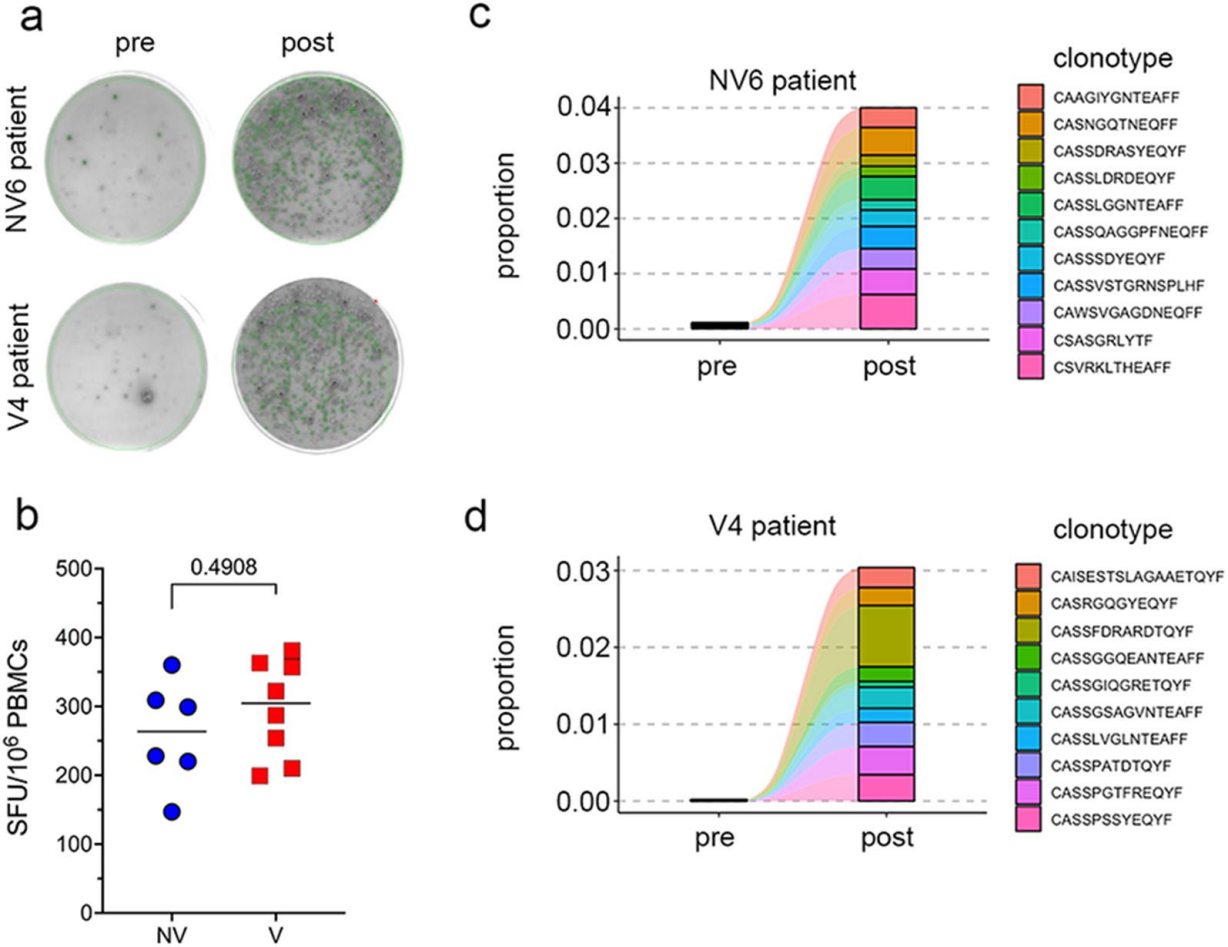
In vitro expansion of S-specific T cells. **a** Representative IFNγ-ELISPOT results for a case of NV and V COVID-19 patient. **b** Frequency of SFU of IFNγ-secreting cells following stimulation with the S-peptide pool in NV and V COVID-19 patient. *p = 0.003, **p = 0.006, Fisher’s test). Statistical comparison was performed by Kruskal–Wallis test. **c**, **d** Representative examples of tracking of the top 10 most abundant clonotypes from the post-stimulation repertoires of NV (**c**) and V (**d**) COVID-19 patients

The in vitro stimulation promotes the expansion of peptide-specific T cell populations, thus allowing the detection of low-frequent clones [20]. To ensure that our S-specific T cell enrichment strategy was effective, we counted the T cell clones responsive to S-peptides using the ELISPOT assay (Fig. 3a, b). Compared to unstimulated PBMCs, those stimulated with S-peptides plus IL-2 showed a higher number of IFNγ-secreting T cell clones (Fig. 3a). The fraction of T cells responsive to S-peptide was not significantly different between V and NV individuals (Fig. 3b).

To further ascertain that post-stimulation TCR repertoires contained S-enriched clonotypes we took advantage of TCRβ sequences with known S-epitope specificity present in the public VDJdb [21] and MIRA [20] databases. The merged the VDJdb/MIRA dataset contained pairings of 26,422 unique TCRβ sequences and 388 S peptides from VDJdb and MIRA databases. The overlap of this dataset with our COVID-19 dataset resulted in 952 unique TCRβ sequences associated with 184 S-epitopes. For each patient, the cumulative frequency of S-specific TCRβ sequences was significantly increased in post-stimulation TCR repertoires (median 7.8; 95%CI 1.4–17.5) than pre-stimulation ones (median 0.8; 95%CI 0.2–1.9%, P < 0.0001, Wilcoxon matched-pairs signed rank test) (Table 2). Furthermore, the 10 most abundant clonotypes of each post-stimulation repertoire were increased in frequency compared to the pre-stimulation frequency (Fig. 3c, d). The S-specific TCRβ sequences accounted for an average of 12.9% (range, 0.7–38.8%) and 10.8% (range, 1.3–29.1%) of the entire post-stimulation repertoires of NV and V group, respectively (Table 2), indicating that the T-cell stimulation with S-peptides was similarly efficient in PBMCs from V and NV patients, in agreement with the ELISPOT results.

### Epitope mapping of S-specific TCRβ clonotypes in COVID-19 repertoires

To further infer the specificity of the TCRβ clonotypes, we performed a clustering of TCRβ sequences based on sequence similarity to S-specific public clonotypes present in the VDJdb/MIRA dataset. To this end, the GLIPH2 algorithm can reliably group TCRs of common specificity from different T-cell samples, organizing clusters of TCR sequences according to their likely antigenic specificities [12, 13, 28].

**Fig. 4:**
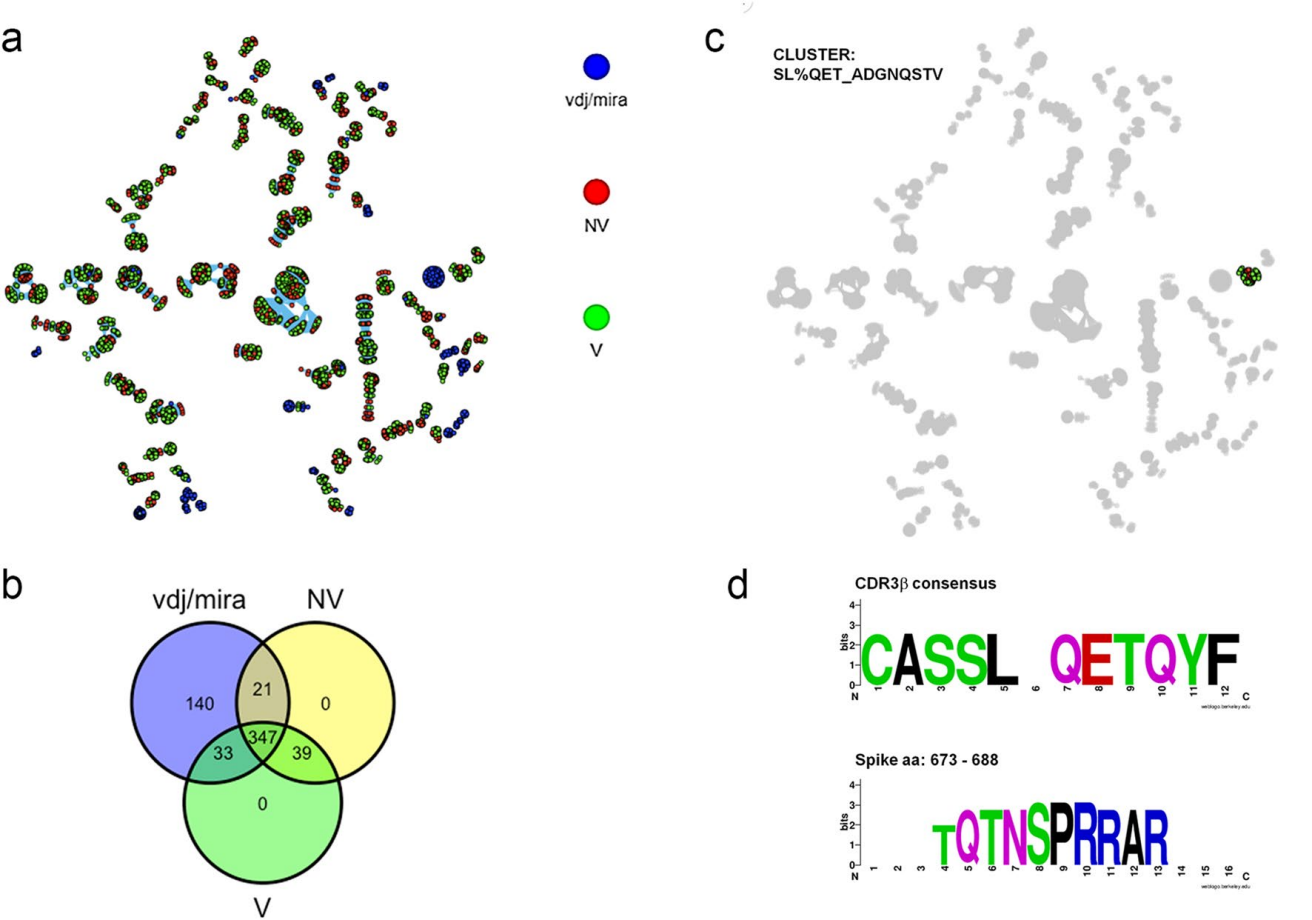
Inference of TCRβ clonotypes specificity by GLIPH analysis (**a**) Network analysis of the most significant (Fisher score < 10^–4^) specificity groups annotated with TCRβ sequences from NV (red), V (green), and VDJdb/MIRA (blue) datasets. Each dot is a specificity group, edges indicate the presence of identical TCRβ sequence(s) shared across two specificity groups. **b** Shared number of S-epitopes in VDJdb/MIRA, V and NV groups. **c**, **d** Characteristics of a representative clustered specificity group, including a representation of amino acid sequence alignment of both the multiple TCRβ in the cluster (**c**), and the associated S region (**d**)

We applied GLIPH2 to a dataset that included our COVID-19 TCRβ clonotypes and the VDJdb/MIRA dataset. We identified 347 specificity groups shared by N, NV, and VDJdb/MIRA datasets, while 21 and 33 specificity groups were shared by NV and VDJdb/MIRA, or V and VDJdb/MIRA datasets, respectively (Fig. 4a, b). The presence of TCRβ sequences from the VDJdb/MIRA dataset in each of the clustered specificity groups allowed us to infer the epitope specificity of the TCRβ sequences from the NV and V datasets within the same cluster [13] (Fig. 4c, d). We identified a total of 1171 TCRβ sequences associated with 149 peptides, of which 130 epitopes were shared between V or NV groups (Table 3). The cluster TCRβ sequences account for an average of 30% of the entire post-stimulation repertoires.

**Table 3:** Characteristics of the specificity clusters obtained by GLIPH analysis

Type^a^	Tag^b^	Size^c^	Fisher score^d^	Members^e^	Epitope^f^	S region (aa)^g^
Local	SPDI_4_22	16	1.20E-27	CASSPDIACTF CASSPDIDQFF CASSPDIEAFF CASSPDIEDFF CASSPDIEQFF CASSPDIEQYF CASSPDIGAFF CASSPDIGLFF CASSPDILAFF CASSPDILDHNEQFF CASSPDILHTF CASSPDINYGYTF CASSPDIQAFF CASSPDITIYF CASSPDITQYF CASSPDIVAFF	YLQPRTFL YLQPRTFLL YYVGYLQPRTF	268—278
Global	%GYNE	8	6.00E-04	CSVDGYNEQFF CSVSGYNEQFF CATSGYNEQFF CASSGYNEQFF CGLAGYNEQFF CASKGYNEQFF CASGGYNEQFF CASRGYNEQFF	SSANNCTFEY VYSSANNCTF	158—169
Global	%RNTE	10	5.20E-06	CSVVRNTEAFF CASIRNTEAFF CSVARNTEAFF CASTRNTEAFF CSVERNTEAFF CASSRNTEAFF CSASRNTEAFF CSLQRNTEAFF CASKRNTEAFF CASGRNTEAFF	YFPLQSYGF HLMSFPQSA YHLMSFPQSA	488–496 1046—1055
Global	R%SYE	10	0.00015	CASRLSYEQYF CGTRVSYEQYF CASRFSYEQYF CASRYSYEQYF CASRHSYEQYF CSARSSYEQYF CSARGSYEQYF CASRDSYEQYF CASRQSYEQYF CASRSSYEQYF	LPPAYTNSF	24–32
Global	S%GET	17	2.70E-06	CSASTGETQYF CASSVGETQYF CAWSVGETQYF CASSAGETQYF CASSTGETQYF CSASLGETQYF CASSLGETQYF CASSIGETQYF CASSFGETQYF CASSMGETQYF CASSRGETQYF CASSQGETQYF CASSHGETQYF CASSEGETQYF CASSYGETQYF CASSDGETQYF CASSGGETQYF	AEIRASANL AEIRASANLA ASANLAATK VQPTESIVRF QPTESIVRF TESIVRFPNI FPNITNLCPF RFPNITNLCPF QYIKWPWYI YEQYIKWPW	1015–1027 319–337 1205–1215
Global	S%GLNTE	8	0.0014	CASSLGLNTEAFF CAWSIGLNTEAFF CASSVGLNTEAFF CASSSGLNTEAFF CASSQGLNTEAFF CASSTGLNTEAFF CATSDGLNTEAFF CASSHGLNTEAFF	KLPDDFTGCV AIPTNFTISV AYSNNSIAIPTNF IPTNFTISV NSIAIPTNF	423–432 705–721
Global	S%GNE	12	0.0015	CASSLGNEQFF CASSLGNEQYF CSASMGNEQFF CASSFGNEQFF CAISFGNEQFF CASSVGNEQFF CASSQGNEQYF CASSQGNEQFF CACSQGNEQFF CASSSGNEQFF CASSRGNEQFF CAISGGNEQFF	FFSNVTWFH FLPFFSNVT LPFFSNVTW PFFSNVTWF APHGVVFL APHGVVFLHV GVVFLHVTY VVFLHVTYV FGEVFNATRF FNATRFASVY GEVFNATRF NATRFASVY	54–65 1055–1067 337–350
Global	S%GNQP	10	0.00024	CASSGGNQPQHF CASSSGNQPQHF CASSFGNQPQHF CASSLGNQPQHF CASSYGNQPQHF CASSVGNQPQHF CASSEGNQPQHF CASSRGNQPQHF CSASTGNQPQHF CASSTGNQPQHF	ASQSIIAYTM RSVASQSII SQSIIAYTM VASQSIIAY	684–696
Global	S%GYE	9	0.011	CASSQGYEQYF CSASQGYEQYF CSASRGYEQYF CASSSGYEQYF CASSEGYEQYF CASSSGYEQFF CASSRGYEQYF CAWSGGYEQYF CASSGGYEQYF	DGVYFASTEK FPQSAPHGV GVYFASTEK FPQSAPHGVVF LPFNDGVYF LPFNDGVYFA VLPFNDGVY GAEHVNNSY IGAEHVNNSY	82–96 1051–1061 650–659
Global	S%SSYE	9	0.0031	CASSESSYEQYF CASSSSSYEQYF CASSQSSYEQYF CAVSKSSYEQYF CASSDSSYEQYF CASSRSSYEQYF CAISESSYEQYF CSASGSSYEQYF CASSGSSYEQYF	CFTNVYADSF FTNVYADSF FTNVYADSFV KLNDLCFTNV LNDLCFTNVY LEPLVDLPI SEPVLKGVKL	385–400 1260–1269
Global	S%STDT	10	0.024	CASSLSTDTQYF CASSVSTDTQYF CASSISTDTQYF CACSASTDTQYF CASSTSTDTQYF CSASGSTDTQYF CASSGSTDTQYF CASSASTDTQYF CSASSSTDTQYF CASSQSTDTQYF	ALDPLSETK CALDPLSETK	290–299
global	S%SYE	17	1.10E-05	CASSFSYEQYF CASSYSYEQYF CAWSLSYEQYF CASSLSYEQFF CASSLSYEQYF CASSISYEQYF CASSRSYEQYF CASSNSYEQYF CASSQSYEQYF CAISESYEQYF CASSESYEQYF CASSSSYEQYF CASSGSYEQYF CSASDSYEQYF CASSDSYEQYF CASSVSYEQYF CASSASYEQYF	TEILPVSMTK CMTSCCSCLK MTSCCSCLK	724–733 1236–1245
Global	S%TYE	10	0.00051	CASSVTYEQYF CASSATYEQYF CASSLTYEQYF CASSQTYEQYF CASSRTYEQYF CASSSTYEQYF CASSHTYEQYF CASSYTYEQYF CASSFTYEQYF CASSGTYEQYF	IYSKHTPINL	203–212
global	S%YE	8	6.40E-05	CASSSYEQYF CSASDYEQYF CASSAYEQYF CASSDYEQYF CASSGYEQYF CASSTYEQYF CASSVYEQYF CASSLYEQYF	APGQTGKIA GQTGKIADY KIADYNYKL QTGKIADYNY RQIAPGQTGK	407–424
Global	S%YNE	18	4.20E-09	CASSSYNEQFF CASSEYNEQFF CASSQYNEQFF CASSDYNEQFF CASSTYNEQFF CSVSGYNEQFF CATSGYNEQFF CASSGYNEQFF CSASSYNEQFF CASSKYNEQFF CASSAYNEQFF CASSRYNEQFF CASSHYNEQFF CASSLYNEQFF CSASLYNEQFF CASSIYNEQFF CASSVYNEQFF CASSYYNEQFF	STQDLFLPFF CTLKSFTVEK TQDLFLPFF SETKCTLKSF TLKSFTVEK	49–58 296–309
Global	SL%ET	13	0.00089	CSASLGETQYF CASSLGETQYF CASSLAETQYF CASSLSETQYF CASSLQETQYF CASSLRETQYF CASSLKETQYF CASSLEETQYF CASSLDETQYF CASSLMETQYF CASSLTETQYF CASSLVETQYF CASSLLETQYF	AEVQIDRLI AEVQIDRLIT VEAEVQIDRL VQIDRLITGR	986–999
Global	SL%GE	12	0.0011	CASSLGGEQYF CASSLAGEQYF CASSLGGEAFF CASSLAGEAFF CASSLSGEQFF CASSLGGEQFF CASSLRGEQYF CASSLEGEQFF CASSLVGEQFF CASSLLGEQYF CASSLVGEQYF CASSLIGEQYF	TEKSNIIRGW GRLQSLQTY LITGRLQSL RLQSLQTYV	95–104 996–1008
global	SL%GET	13	6.60E-06	CASSLTGETQYF CASSLAGETQYF CASSLVGETQYF CASSLSGETQYF CASSLNGETQYF CASSLFGETQYF CASSLYGETQYF CASSLLGETQYF CASSLWGETQYF CASSLIGETQYF CASSLEGETQYF CASSLRGETQYF CSASLQGETQYF	LLFNKVTLA	821–829
Global	SL%GNE	12	9.70E-06	CASSLAGNEQFF CASSLTGNEQFF CASSLVGNEQYF CASSLSGNEQFF CTSSLAGNEQYF CASSLVGNEQFF CASSLSGNEQYF CASSLGGNEQFF CAISLEGNEQFF CASSLEGNEQFF CASSLLGNEQFF CASSLLGNEQYF	KTSVDCTMYI	733–742
Global	SL%GNTE	12	0.00049	CASSLGGNTEAFF CASSLAGNTEAFF CASSLSGNTEAFF CASSLNGNTEAFF CASSLVGNTEAFF CASSLTGNTEAFF CASSLQGNTEAFF CASSLEGNTEAFF CASSLRGNTEAFF CASSLDGNTEAFF CASSLIGNTEAFF CASSLLGNTEAFF	LLLQYGSFC LLQYGSFCT CNDPFLGVY CNDPFLGVYY FCNDPFLGVY	135–144 752–760
Global	SL%GTE	9	0.00057	CASSLEGTEAFF CASSLSGTEAFF CASSLRGTEAFF CASSLSGTEQFF CASSLGGTEAFF CASSLTGTEAFF CASSLVGTEAFF CASSLLGTEAFF CASSLIGTEAFF	LLTDEMIAQY LTDEMIAQY LTDEMIAQYT VLPPLLTDEMIAQY	860–873
Global	SL%LNTE	11	7.90E-06	CASSLGLNTEAFF CASSLALNTEAFF CASSLNLNTEAFF CASSLSLNTEAFF CASSLVLNTEAFF CASSLTLNTEAFF CASSLKLNTEAFF CASSLQLNTEAFF CASSLELNTEAFF CASSLRLNTEAFF CASSLDLNTEAFF	GYQPYRVVVL PYRVVVLSF QPYRVVVL QPYRVVVLSF	504–513
Global	SL%QET	8	0.0039	CASSLNQETQYF CASSLGQETQYF CASSLSQETQYF CASSLDQETQYF CASSLQQETQYF CASSLTQETQYF CASSLAQETQYF CASSLVQETQYF	QTNSPRRAR SPRRARSVA TQTNSPRRAR SYQTQTNSPR	673–688
Global	SLG%E	15	3.10E-05	CASSLGNEQFF CASSLGGEQYF CASSLGNEQYF CASSLGSEQYF CASSLGDEQFF CASSLGTEAFF CASSLGGEAFF CASSLGHEQYF CASSLGGEQFF CASSLGREQFF CASSLGDEQYF CASSLGYEQYF CASSLGVEQFF CASSLGIEQYF CASSLGIEQFF	KVFRSSVLH VYYPDKVFR YPDKVFRSS YPDKVFRSSV AENSVAYSN AENSVAYSNN LGAENSVAY	36–49 699–710
Global	SLG%NTE	10	0.00035	CASSLGQNTEAFF CASSLGHNTEAFF CASSLGRNTEAFF CASSLGMNTEAFF CASSLGENTEAFF CASSLGSNTEAFF CASSLGLNTEAFF CASSLGVNTEAFF CASSLGGNTEAFF CASSLGANTEAFF	FTISVTTEIL KEIDRLNEV	718–727 1181–1189

^a^Type of cluster, where “global” similarity referes to CDR3 differing up to one amino acid, and “local” refers to shared enriched CDR3 amino acid motifs (> tenfold-enrichment, probability < 0.001) [12];

^b^Patter of the cluster; global pattern contains '%', which indicates position allowing variants; local pattern starts with 'motif-Fisher_score estimates the bias of the pattern presenting in target data versus reference data using Fisher exact test;

^c^Number of distinct CDR3s included in the cluster;

^d^Fisher_score estimates the bias of the pattern presenting in target data versus reference data using Fisher exact test [12];

^e^CDR3 amino acid sequences of clonotypes from the COVID-19 dataset; the clonotypes from VDJdb/MIRA dataset;

^f^S-peptides associated with clonotypes of the VDJdb/MIRA dataset included in the cluster;

^g^Amino acid position in the spike protein (uniprot ID P0DTC2)

**Fig. 5:**
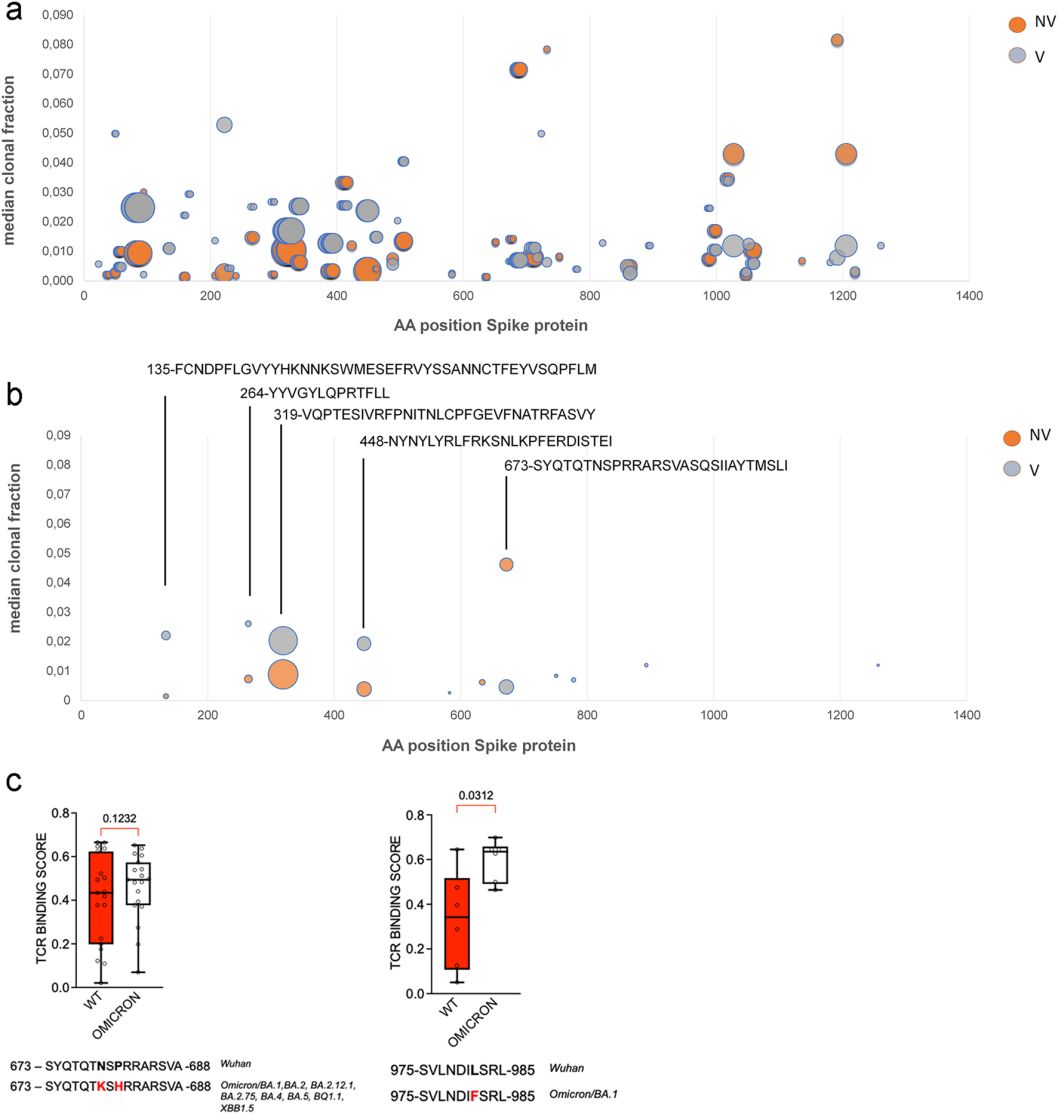
Mapping of TCRβ associated with S epitopes as resulted from GLIPH2 analysis. **a** Median clonal fraction refers to the median of clonotype frequencies from the individual pre-stimulation repertoires. Dimension of bubbles correlates with the number of distinct clonotypes associated with the epitope. **b** Glimpse of panel A highlighting the S regions (overlapping epitopes) associated to TCRβ differentially abundant between COVID-19 groups. **C** TCR binding prediction of S peptides from original Wuhan-Hu-1 strain and VOCs variants. The distinct TCRβ sequences associated with the indicated S peptide (Table 3) were evaluated in-silico for predicted binding affinity by the ERGO tool (Mann Whitney test)

Among the 130 shared peptides, TCRβ clonotypes associated with S regions S135-177, S264-276, S319-350 and S448-472 were significantly more abundant in the post-stimulation repertoires of V group than the NV group (Fig. 5a, b and Table 4). Conversely, TCRβ clonotypes associated with protein region S673-699 was significantly more abundant in the post-stimulation repertoires of NV group than the V group (Fig. 5a, b and Table 4). Moreover, the S645-645 and S751-760 peptides were exclusively associated with TCRβ clonotypes in the NV group, while S778-789, S863-871 and S1260-1269 peptides were exclusively associated with TCRβ clonotypes in the V group (Fig. 5a, b and Table 4). These results indicate a distinct profile of TCR epitope specificity between the N and NV COVID-19 groups following infection with SARS-CoV-2.

**Table 4:** Summary of TCRβ associated with spike peptides. In bold, the data represented in Fig. 5b are highlighted

aa Position^a^	**U**nvaccinated			Vaccinated			p value^e^
	n. clonotypes^b^	Median enrichment^c^	Median clonal fraction^d^	n. clonotypes	Median enrichment	Median clonal fraction	
23–59	55	15.8	0.0063	13	8.8	0.0162	0.1589
82–94	9	12,1	0.0115	73	15.0	0.0203	0.3630
**135–177**	**20**	**11.3**	**0.0015**	**11**	**9.8**	**0.0222**	**0.0116**
207–240	10	8.4	0.0021	9	8.8	0.0159	0.1834
**264–276**	**16**	**19.8**	**0.0025**	**5**	**8.4**	**0.0261**	** < 0.0001**
**319–350**	**118**	**11.2**	**0.0051**	**108**	**8.8**	**0.0203**	** < 0.0001**
385–423	41	16.5	0.0039	58	8.6	0.0187	0.7000
**448–472**	**30**	**8.7**	**0.0039**	**26**	**10.3**	**0.0194**	** < 0.0001**
488–506	21	8.8	0.0094	10	9.3	0.0296	0.071
582–591	1	8.0	0.0027	0			–
645–645	5	17.9	0.0062	0			–
**673–699**	**24**	**15.5**	**0.0041**	**29**	**15.5**	**0.0004**	0.0013
705–732	25	18.2	0.0191	17	8.7	0.0156	0.6349
751–760	2	9.6	0.0084	0			–
778–792	0			3	9.9	0.0070	–
859–864	16	8.8	0.0039	16	8.8	0.0050	0.2016
893–902	0			2	8.4	0.0120	-
986–999	18	8.6	0.0088	12	8.9	0.0176	0.2381
1015–1027	15	74.2	0.0352	14	10.3	0.0266	0.6230
1046–1059	21	15.3	0.0074	16	13.2	0.0061	0.1289
1260–1269	0			1	14.7	0.012	–

^a^Amino acid position in the spike protein (uniprot ID P0DTC2)

^b^Number of clonotypes associated to epitope within the indicated spike region; showing a post/pre frequency > 8

^c^Median of the enrichment (post/pre stimulation ratio of clonal fration) showed by the clonotypes associated to epitope within the indicated spike region

^d^Median of the pre-stimulation clonal fration showed by the clonotypes associated to epitope within the indicated spike region

^e^Statistical differences were evaluated by Mann–Whitney test

### Effect of epitope mutations on TCR-peptide binding affinity

The T cell expansion procedure was performed using ancestral Wuhan-Hu-1-derived S peptides to stimulate PBMCs from Omicron/BA2, BA.4, and BA.5-infected patients. One potential concern was that TCR specificity might vary between individuals exposed to both the Wuhan-Hu-1 and Omicron antigens (group V) versus those exposed only to the Omicron antigen (group NV).

Among the relevant S peptides of the ancestral Wuhan-Hu-1 strain, the S673-688 and S975-985 were subjected to amino acid substitution within the variants BA.1, BA.2, BA.2.12.1, BA.2.75, BA.4, BA.5, BQ1.1, XBB1.5. We addressed the impact of amino acid substitution on TCRβ binding by performing an in-silico analysis using ERGO, a reliable and robust tool for TCR-peptide binding affinity prediction and classification [22]. ERGO-II results indicated that the variant in the S975-985 region (S981L > F, of Omicron/BA.1) increased the peptide binding probability score of TCRβ clonotypes from both N and NV groups, while the variant in the S673-688 region (S679N > K, S682N > K of Omicron/BA2, BA.4, BA.5, BQ1.1 variants) did not affect the peptide binding probability score of TCRβ clonotypes associated with this region (Fig. 5c). These results suggested that epitope mutations did not preclude cross-reactive recognition by TCRβ clonotypes of our groups.

## Discussion

In this study, we first highlighted the differences in the general characteristics of the TCRβ repertoire between individuals with COVID-19 who were either vaccinated or unvaccinated. Then, we specifically examined the S-specific TCRβ repertoire, identifying differences that may be important in predicting disease progression and cross-reactivity towards viral variants.

The ability of adaptive immunity to achieve effective TCR diversification may determine the chances of improved outcomes and immune control in infectious and cancer diseases [29–32]. A study on the T cell repertoire in SARS-CoV-2 infected patients showed that TCR diversity may influence disease outcomes, and that patients with asymptomatic or mild clinical infection have a highly diversified TCR repertoire, while patients with severe COVID-19 have a less diverse TCR repertoire [15]. Consistent with this previous observation, we found that patients with SARS-CoV-2 exhibited lower diversity and higher clonality compared to the healthy control group, which is expected in the presence of a productive infection. Furthermore, COVID-19 patients with breakthrough infections after vaccination, all of whom had a mild clinical course, showed a trend towards greater diversity compared to non-vaccinated COVID-19 patients, although this difference did not reach statistical significance. Additionally, the distribution of diversity indices within the two groups appeared different, with much more homogeneous diversity values in vaccinated patients compared to the unvaccinated ones. It is possible that in patients with breakthrough infections, the presence of established immunological memory from vaccination allowed for a more expansion of S-specific T cells, resulting in a repertoire with a greater and homogeneous diversity compared to the unvaccinated group. It should be noted that the two patients who developed severe COVID-19 exhibited very narrow true diversity and high clonality, consistent with the previous referenced studies [15].

The analysis of Vβ gene segments distribution within the TCRβ repertoire showed that TRBV7-2, TRB29-1, and TRBV30 Vβ gene segments had a significantly higher clonal fraction than those observed in the unvaccinated group. T cells expressing the TRBV7-2 gene segment have been associated with various clinical conditions, including immune response to human rhinovirus infection and autoimmune diseases such as multiple sclerosis and rheumatoid arthritis [33, 34]. Our results further expand the conditions associated with TRBV7-2 Vβ gene segment and support further investigations to uncover the clinical significance of this association in COVID-19.

The S673-699 region was specifically linked to TCRβ clusters in the unvaccinated group, among the S regions that were differentially associated with TCRβ from vaccinated or unvaccinated groups. Some researchers have suggested that this region may have super antigenic properties, which could potentially contribute to the severe immune response observed in some COVID-19 patients, leading to cytokine storms and multiorgan failure [35–37]. Superantigen-mediated T cell expansion occurs through a less specific interaction with TCRs, leading to the activation of a large proportion of the T-cell population [38]. Although the super antigenic character of the S673-699 region is still being investigated, it is possible that the efficacy of the mRNA COVID-19 vaccines in preventing severe disease and death is also due to reduced exposure to the super antigenic viral determinant, through neutralizing antibodies or reduced viral replication. We also found that TCRβ clonotypes associated with S regions S135-177, S264-276, S319-350 and S448-472 were significantly more abundant in the vaccinated group than in the unvaccinated group. There could be several reasons for this difference, which are likely related to the various ways in which these peptide regions are expressed and presented to the immune system between vaccination and natural infection. In our small cohort study, the presence of specific clonotypes associated with these S regions was found to be linked with a milder course of the disease. Therefore, detecting T cells that are specific to these regions may have a positive impact on disease prognosis and could potentially be used as a prognostic tool.

Our study obtained TCRβ sequences specific for the S protein from PBMCs of patients infected with the Omicron/BA2, BA.4, and BA.5 variants, after stimulation with a pool of S peptides derived from the ancestral Wuhan-Hu-1 sequence. In vitro expansion of these cells indicated that TCRβ cross-reacted with peptides from the ancestral strain. Our in-silico analysis using the ERGO tool showed that the variant in S975-985 region increased the probability score of TCRβ clonotype peptide binding in both vaccinated and unvaccinated groups, while the variant in the S673-696 region did not affect the probability score of peptide binding of TCRβ clonotypes associated with this region. These findings suggest that TCRβ clonotypes from both groups can recognize cross-reactive epitopes despite mutations.

The study provides TCRβ sequences, which is a valuable information for understanding the immune response. However, it is important to note that the lack of alpha chain information represents a limitation to the study. Indeed, the alpha chain plays a critical role in shaping the T cell receptor’s specificity and affinity for antigen recognition. However, although the absence of alpha chain information may hinder the interpretation of epitope specificity, this limitation is diminished by the fact that the prediction algorithms we used were predominantly trained with TCRβ data. Moreover, the potential confounding factors in our study, particularly in relation to the small and diverse sample size, limit the ability to draw any association of TCR signature with clinical variables. The presence of both immunocompetent and immunodeficient patients, the limited number of severe cases (n = 2), and variations in vaccination status and HLA types indeed pose challenges in interpreting the TCR data with high precision. Moreover, we recognize that the limited scope of our sample size, comprising only 14 individuals, restricts our ability to generalize our findings to a broader population. This sample size limitation is especially pertinent given the complexity of TCR repertoires and their interactions with various clinical variables. Our study's focus on selected aspects of the immune response further narrows the scope of our findings.

## Conclusions

Overall, our findings reveal significant differences in TCR specificity between natural and breakthrough infections and identified unique TCR signatures associated with disease severity, providing insights into the potential factors influencing clinical outcomes.

### Supplementary Information

**Additional file 1.** Immune repertoire data.

## Data Availability

All the data was included in the manuscript and additional file. All the materials and reagent sources used in this study are described in the methods section.

## Abbreviations

CDR3: Complementarity-determining region 3
ELISPOT: Enzyme-linked immunoSpot
IFNγ: Interferon gamma
NV: Not-vaccinated
PBMCs: Peripheral blood mononuclear cells
S: Spike protein of SARS-CoV-2
SFU: Stimulating forming unit
TCR: T cell receptor
V: Vaccinated
VoCs: Variants of concern
